# In Search of Manifestations and Imaginations of the Climate Crisis: A Longitudinal Analysis of an Online Diary

**DOI:** 10.1007/s12124-025-09941-4

**Published:** 2025-10-01

**Authors:** Maeva Perrin, Oliver Clifford Pedersen

**Affiliations:** 1 Faculty of Psychology and Education Sciences, University of Geneva, 1211 Geneva, Switzerland; 2 Institute of Psychology and Education, University of Neuchâtel, 2000 Neuchâtel, Switzerland

**Keywords:** Climate crisis, Imagination, Futures, Eco-anxiety, Longitudinal

## Abstract

Crises are not all sudden and spectacular – some are incremental and manifest in unlikely places. We explore how one person’s experience and imagination of the climate crisis developed across 24 years of online diary writing. This longitudinal analysis captures the subtle shifts in people’s experience close to real time, allowing us to theorise when and how the climate crisis is manifested, felt, and imagined. We move beyond the common definition of crisis as an exceptional disruption in time, recognising that it can also be a slow and elusive process, and instead ask which events bring the climate crisis to the forefront of people’s experience. The analysis highlights three periods with imaginative transformations, detailing a temporal realignment and gradual differentiation. Moving from a distant cloud on the horizon into the present, the diarist’s imagination increasingly gains concrete and apocalyptic form. In tandem, we trace fluctuations in eco-emotions to challenge static and linear categorisations, suggesting that the dichotomy between indirect and direct manifestations is intimately entangled through time and scaffold the imagination. Our research attempts to provide a dynamic and contextually sensitive approach to study and understand how the climate crisis is experienced and imagined over time.

## Introduction

Crises are increasingly part of the public, political, and scientific discourse, yet research is often not sensitive to when people experience and imagine them, nor how they evolve with time. This potential blind spot partly stems from prevailing notions of *crisis* and the practical difficulties in following crisis experiences over an extended period. The quintessential definition of *crisis* describes a temporary state of exception that demands a decision before a new equilibrium emerges (Koselleck & Richter, 2006). However, researchers have begun to question the “benign universe” assumption ingrained in this definition, as it does not do sufficient justice to the millions of people living in states of chronic or endemic crisis (Vigh, 2008). When studies focus on time-bound and politicised societal events that are labelled as crises, the slow and invisible forms of violence, often displaced in time and space, may remain out-of-sight (Nixon, 2011). Inspired by others who argue that focusing on people’s experiences (e.g. Fassin, 2022) can sensitise research to different forms that crises take and where they might emerge, we explore one diarist’s experiences and imaginations related to the climate crisis across two decades of journaling. Specifically, we intend to explore when and how the incremental, human-caused climate change manifests, and how these manifestations shape imagined climate futures.

We aim to nuance the common approaches to studying crises, recognising that they can also be slow and elusive, by proposing a longitudinal approach. This approach is sensitive to the types of events or information that bring the climate crisis to the forefront and how imagined climate futures are transformed accordingly. While we selected the climate crisis for its slow-building and diffuse nature, this approach can also tease out other, sometimes entangled crises that are obscured when starting from a third-person perspective (Zittoun et al., 2023). We adopt a sociocultural psychological perspective that views people’s experience of crisis as unique, and therefore any crisis appears and manifests differently depending on life trajectories and circumstances. Following people long enough for crisis experiences and imaginations to surface spontaneously calls for longitudinal methodologies. More conventional methodologies, such as ethnography and qualitative interviewing, can capture crisis experiences but remain limited by their shorter (and often predefined) duration and elicitation of retrospective and pre-narrativized accounts. Online diaries written for 20 + years offer a curated window into people’s experiences, in close to real time (Zittoun & Gillespie, 2018), without relying on after-the-fact rationalisations.

We first introduce the sociocultural psychological model of imagination, used as a prism to analyse how people’s changing experiences shape imagined climate futures, and then briefly discuss how the notion of crisis has been conceptualized more broadly. We revisit existing connections between imagination and the climate crisis in psychological literature, such as eco-anxiety, to propose a more dynamic and content-oriented approach (Pedersen, in prep.). Subsequently, we venture into the depths of Oswego’s diary to explore where the climate crisis emerges in his writing. We divide the analysis into three distinct periods, each roughly indicating a significant imaginative transformation.

## Imagination and Crisis on the Move

Imagination is a psychological and social process through which individuals and collectives can imagine (alternative) futures and potentially enact change (Hawlina et al., 2020). The process is triggered by an event, is nourished by different symbolic resources – such as books, films, political discourses, social norms, etc. – and can lead to new forms of thinking and acting (Zittoun & Gillespie, 2016). As Zittoun (2006) explains, symbolic resources are cultural elements intentionally mobilized by people to make sense of their experience. That said, people’s capacities for imagining are not evenly distributed (Pels, 2015; Stoetzler & Yuval-Davis, 2002) and therefore demand sensitivity to who imagines what, and for whom. Various sociocultural and material factors establish boundaries for the imaginable – also called imaginative horizons (Zittoun et al., 2020). Imagination is steeped in power relations that taints both individual and collective imagination and can function both as an instrument of oppression and act of resistance. Migration regimes and caseworkers structure often constrain people’s imagination (Power & Botelho, this issue; Crafter et al., this issue), whereas Afrofuturism excellently shows the resistance towards a future overdetermined by foreign dystopian predictions (Eshun, 2003). Individual and collective imagination are therefore inextricably related, through the resources that crystallise and nourish the process. We aim to explore imaginative changes connected to different manifestations of the climate crisis – the latter act as a trigger, although it can also become a resource that nourishes the process.

Before discussing the burgeoning psychological research that addresses connections between the climate crisis and imagination, we will first briefly elaborate on crisis more generally. Crisis is often defined as an exceptional moment marking a rupture in a previously established order, implying clear beginnings and endings (Fassin, 2022). Research has recently begun to pay greater attention to the endemic (Vigh, 2022), repeated (Pedersen, 2023), and slow (Ahmann, 2024) crises. Acknowledging that crises can be endemic rather than episodic warrants a closer examination of what temporalities, intensities, visibilities, and scales define each crisis (Bergman-Rosamond et al., 2022). Crises often heighten uncertainties (Knight & Stewart, 2016) and erode imaginations as temporal expectations become obsolete – contingent on the crisis. For example, when a crisis has an anticipated endpoint, the uncertain might be less oppressive for those who can “wait out the storm” (Pedersen, 2023), while chronic crises might shift people’s temporal orientation towards the immediate because no end-point is in sight (Vigh, 2008). The characteristics of a crisis are therefore important for what will be imagined. Stenner (this issue) proposes that liminal occasions (such as crisis) are moments when prior structures of meaning are suspended, opening a paradoxical space – unsettling yet fertile – for imagination and transformation. Most crisis research does not offer a longitudinal perspective that is sensitive to how crises are manifest, experienced, and imagined over time.

Researchers have begun discussing the associated “imaginative” challenges. For example, Moore and Milkoreit (2020) described a “triple failure of the imagination” related to the climate crisis’ because the risks are difficult to grasp in their entirety. They emphasise the absence of collective and detailed imaginations for a viable future, along with a failure to imagine explicit pathways to achieve it. Paradoxically, there has simultaneously been a surge in disaster stories and climate fiction demonstrating imaginative attempts, often picturing future catastrophes. While such stories can humanise the abstract (Milkoreit, 2016), studies indicate that these tend to offer survivalist accounts (Dasilva, 2019), that is, how life in post-apocalyptic might be, without offering viable solutions to prevent the apocalypse. Climate messaging also plays an important role (see Lamb et al., 2020 for an overview), swaying the content of imagined futures. For instance, positive messaging appears unrealistic when coupled with low trust in governments or if the threat seems insurmountable, fear-based messaging can lead to paralysis, despair, or climate fatigue (Troy et al., 2024). Recognising fear’s potentially paralysing effect, some climate movements have adopted more hope-based messaging, focusing on desirable outcomes and fostering a sense of possibility (Friberg, 2022). Others argue for a post-apocalyptic environmentalism in which hope is regained by acknowledging pain, voicing loss, anger or despair, igniting new imagination away from naïve optimism (Cassegård & Thörn 2018). In contrast, big oil corporations’ insidious campaigns to instil doubt (Maani et al., 2022) aim to offer an alternative imagination that maintains the status quo. Historical analogies can feed into imaginations of the future (Wagoner, this issue). Here, different symbolic resources – whether narratives of survival, campaigns, or historical analogies – assist people’s pre-imagination or anticipation of crises (Gillespie and Zittoun, this issue), and when these are no longer adequate, ruptures may arise.

The scale and speed required to address the climate crisis also strain the imagination (Troy et al., 2024), especially when considering the short-termism of individuals and the systems’ temporal horizon – how far into the future imaginations stretch (Hessen et al., 2024). People’s concern is often limited to their own lifespan, or that of their children. This temporal horizon, combined with incrementality, can create a psychological distance making the consequences of the climate crisis seem displaced in time and space (Hessen et al., 2024; Nixon, 2011). The crux of the issue is that, on one hand, people’s imagination is overdetermined by apocalyptic stories and, on the other hand, not having the symbolic resources to imagine the necessary preventive measures due to the crisis’ characteristics. The climate crisis can appear as ephemeral because of its incremental and impalpable nature, making the imaginations less urgent and concise. Not having clear imaginations of alternative futures can hamper the ability to engage in transformative change (Milkoreit, 2017), as Norgaard writes, “ [i]magination is power especially in a time of crisis” (2018, p. 171).

One of the major strands of psychological research joining the climate crisis and imagination relates to climate and/or eco-anxiety. Both are concerned with the future as Pihkala writes, anxiety “is future-oriented and related to a threat about which there is significant uncertainty” (2020, p. 2). We will refer to eco-anxiety as an umbrella term. Meta-analyses link eco-anxiety with adverse health outcomes such as depression, stress, and insomnia (Boluda-Verdú et al., 2022). Others warn against pathologizing eco-anxiety which can propel sustainable action, especially when the “right” balance between anxiety and hope is maintained (Pigott, 2018; Sangervo et al., 2022). When eco-anxiety fosters sustainable behaviours, it becomes a “practical anxiety” (Clayton, 2020; Kurth & Pihkala, 2022). Ultimately, the form eco-anxiety takes depends on myriad factors as indicated by demographic studies (Wullenkord et al., 2021). While this line of research offers important insights into emotional responses to the climate crisis (Kurth & Pihkala, 2022), studies tend to offer a snapshot of these emotions and ignore the content of the futures that induce anxieties and related emotions (Pedersen, in prep.). Our longitudinal approach shows how these emotions fluctuate with time, respond to specific events and developing imaginations.

We are aware that research, including climate research, from the Global North is criticised for not breaking with capitalist extractivism (Descola & Pignocchi, 2022), human exceptionalism (Haraway, 2016), and colonial temporalities (Death, 2022). For example, indigenous communities are already living in dystopian realities, marked by centuries of colonialism and ecological collapse (Whyte, 2017). These criticisms showcase the ideological force of dominant imaginaries that portray the collapse as being on the horizon and reflect vast inequalities in terms of whose experiences and imaginations are heard. This point is also present in the question of which words to use, since *crisis* and *emergency* carry temporal assumptions and urgencies (Clot-Garrell, 2024). Shifting temporality can also mark the transition from eco-anxiety to eco-grief (Ágoston et al., 2022; Cunsolo et al., 2020). These critiques emphasise the need to be sensitive to how different populations experience and imagine the climate crisis, and we propose that our approach can potentially also address some of those.

Imagination clearly occupies a central role in how people experience the climate crisis. As detailed, this link is relevant to subjects from climate fiction and eco-emotions to social movements. However, instead of assuming what people imagine at one moment in time, we aim to understand how their imagination develops, and the resources it draws on. We hope this approach might reveal inequalities in what can be imagined, highlight the importance of people’s trajectories, and make research sensitive to the content of imagined climate futures and the events and information that nourish them. Having a concrete and actionable imagination is important for people’s ability to act (Wright et al., 2020), and disentangling these may nuance current understanding of the growing eco-anxieties many feel.

## Following the Pen Through the Storm

We turn to online diaries to theorise where and how crises manifest and to maintain sensitivity to the impalpability and temporally murky crises, which are difficult to follow methodologically. Diaries are suited for following the spectacular and mundane manifestations of crises, and to theorising the symbolic resources that nourish imaginations across time. These documents are curated windows into people’s experiences and processes of sensemaking (Zittoun et al., 2023), are usually written close to real time, and can therefore capture twists and turns concealed by other methodologies, such as retrospective interviewing. Instead, diaries capture lives lived forward, with all the doubts and uncertainty related to not knowing where the story ends. This open-endedness and long timespan make diaries pertinent for studying emerging crises and their transformations. To illustrate this methodological proposition, we decided to focus on the climate crisis, rather than completely looking for crises inductively. This strategy reflects the fact that the climate crisis has been a slow-burn for decades – at least for the privileged. We hope that following the climate crisis in people’s writing can potentially provide a new account of what form it takes, and what climate futures are imagined through time.

Among the 41 diarists who participated in the research, we decided to focus on a single diarist, Oswego, because he has written about the climate crisis semi-regularly since 2000. Twenty-four years of writings provide ample opportunity to analyse the diarist’s imagination longitudinally. Oswego’s diary contains 1,369 entries and 858,515 words in the period between 1999-05-11 and 2024-11-05. Since much of his writing does not relate to the climate, we used natural language processing to filter the diary. Specifically, we first created a generic list of words related to the climate crisis, e.g. “global warming” and “co2 emissions”, to extract all diary entries containing those words using SpaCy. We iteratively assessed how different words fitted and further refined the list by reading through sections of his diary to add words he organically used when writing about the climate crisis. After a few iterations, we landed on 48 entries and 99 word occurrences.

In addition, we also visited Oswego twice and conducted brief stints of ethnography and qualitative interviews with him to supplement his writing. This material offers thicker descriptions to substantiate the process of theorisation, such as learning about experiences not presented in the diary and getting a glimpse into his daily life and surrounding environment. While Oswego has provided written consent for all parts of the research process, we decided to adopt a more processual ethical stance (Mattingly & Throop, 2018). Meeting Oswego was the first (and small) initiative attempting to reduce the vast asymmetries associated with having access to such an intimate account of his life, while he barely had any information about us. Ever since our first meeting, we have kept in touch with Oswego, and still frequently exchange emails about the research, state of the world, our lives, and photographs. Furthermore, the power of analysis and interpretation also rests with us (Descola & Pignocchi, 2022) – an asymmetry apparent when we impose conceptual frameworks onto his life. We therefore shared this and other articles with Oswego before submission to ensure he felt comfortable with how his story is conveyed and to offer him the opportunity to comment. This act represents another small step towards ensuring that his consent extends beyond signing a document years ago – we want to avoid the pitfall of considering consent as a carte blanche. The fact that Oswego is part of the audience (Cornish, 2020) also continuously reminds us not to let the pen flow too freely, as Scheper-Huges (2000) warned, by being aware of what we write, how we write, who might read the article, and what the possible macro- and micro-ethical implications might be (Brinkmann & Kvale, 2005).

## Between Near and Far: Imagining the Climate Crisis

Throughout the analysis, we will primarily use his diary to gauge where the climate crisis manifests for Oswego, that is, identify what events or resources bring him to write about it. In addition, we attempt to detail how various manifestations impinge on his imagination, becoming symbolic resources, particularly to supplement existing research with a longitudinal perspective and explore what form the future takes over time. We give particular attention to the nature of the different events and resources, for example, if they are direct (e.g. experiencing a devastating hurricane first-hand) or indirect (e.g. reading about forest fires in Wyoming) (e.g. Doherty & Clayton, 2011). While different in nature, these can all become symbolic resources nourishing the imagination and we hope to explore if they carry different weight, inspired by studies examining the connection between the form of exposure and people’s psychological distress and sustainable actions (e.g. McDonald et al., 2015). However, instead of operating on a dichotomy, our longitudinal analysis demonstrates how different exposures interact over time according to the person’s unique circumstances. We also attempt to explore whether the resources or events are incremental or sudden, based on the assumption that the slower and less spectacular manifestations tend to be less visible. We have structured the analysis chronologically and split the text into three loosely defined phases that reflect an imaginative shift in terms of how apocalyptic Oswego’s climate future becomes and how temporally close it is.

Oswego first wrote about the climate crisis in 2000, after reading a book by environmentalist Rachel Carson, stressing that humans are the main culprits:

2000-10-30: It is hard for me to overcome a lifetime of exposure to, and knowledge of, man’s destruction and desecration of the natural world around him. On and on it goes. But that child that Carson brought to the ocean’s edge is the future. He represents the hope that we can slow down, if not halt, our injurious ways of living in the world, gobbling up the Earth’s resources and polluting the air and water and soil.

This journal entry begins with a scene from Rachel Carson’s book, in which she brings a toddler to witness violent waves along the Pacific coast. While we do not know if he was reading the book exactly at the moment of writing, his imagination oscillates between fearing that the destruction will go “on and on” and finding a hope imbued in the young child. The parable is that instilling an appreciation for nature at an early age may lead people to become “environmentalists” and “defenders of wildlife” – those who can reverse humanity’s current trajectory. The book functions as a (distant) symbolic resource that supports Oswego’s hope in the future generation. This entry also reveals, and indeed foreshadows the analysis, how different manifestations of the climate crisis (“exposure to” and “acknowledgement of”) co-exist and probably interact. We assume that different manifestations may become different resources for imagining, and grasping these nuances and interactions might contribute to understanding how people imagine climate futures.

### “I think the chickens are coming home to roost”

The first period revolves around the idea that humanity’s actions will have consequences, as the header implies, while still being located on the horizon. It also showcases how direct and indirect extreme weather events become manifestations of the climate crisis for Oswego, triggering imaginative processes. These events condense and spectacularise a largely slow-building and invisible climate crisis, momentarily pulling the distant future into the present, and reflect colloquial understandings of crisis discussed above. They also serve as warnings of what might be to come, hence acting as symbolic resources that nourish imagined climate futures. Throughout this period, Oswego’s imagination remains relatively abstract and distant, as the disaster still looms sometime in the future. Extreme weather events first entered his diary when he saw forest fires blazing over the Bitterroot Mountains in 2001 on the news:

2001-12-13: When fires spread over the Bitterroot Mountains of Montana in early autumn 2000, we read about the conflagration and saw some black and white pictures in the newspapers, or we happened to watch film clips on CNN or the evening news. But nothing can compare to the picture below in capturing the full intensity of those phenomena of Nature -- forest fires -- devastating and yet not surprising given the way humans have altered and interfered with the natural order of fires and climate change during the decades of the 20th century. Never have such terrible fires raged over our drier and drier Western forests than in recent years. […] In the midst of that raging fire, there is always sanctuary, and from water, the symbol of life, comes hope.

Newsreels of forest fires thousands of kilometres away caused Oswego to lament humanity’s role in interfering with the “natural order”, although without conceding hope because the situation is still appraised as evitable (Badaan et al., 2020). Watching the coverage from afar is an indirect, yet spectacular, manifestation that brings the climate crisis into focus but without triggering a radical re-imagination, likely because a collapse remains temporally distant and avoidable.

A few years passed before he wrote about the climate again, until Hurricane Katrina brought the climate crisis back into focus. The hurricane devastated New Orleans in 2005, and the news coverage and imagery had a significant impact on him because he grew up in the city:

2005-12-30: This has been a very disturbing year for me since Katrina almost destroyed my hometown of New Orleans. The weeks and months go by, but the pain and loss are still felt keenly. Although I now live many miles away, it was still home to me for years and nothing can diminish that fact. Something about that terrible storm, months after the tsunami hit south Asia, made me feel very mortal and very vulnerable. 2005 with its fears of bird flu pandemics, endless wars and terrorism in Iraq and the Middle East and elsewhere, riots in Paris, continuing starvation in Africa -- it all brought to mind the precariousness of our existence. Don’t get me started on global warming. That could be the most dire scenario of all.

The destruction lingered with Oswego, as is noticeable in how often he wrote about the disaster afterwards. Similar to forest fires, the hurricane is an extreme weather phenomenon that Oswego experienced indirectly, but his attachment to the city enhanced the significance, reflected in his exclamation: “it is still home to me”. Here, the sharp distinction between direct and indirect manifestations fails to adequately represent Oswego’s experience. An indirect manifestation is overcharged due to an emotional attachment – the event gains a personal character as the streets where his childhood memories unfolded were submerged under water. It resonates with other global crises (Pedersen et al., submitted), yet the climate crisis dwarfs their severity, as he writes: “don’t get me started on global warming”. These global crises culminate into a sense of precarious existence with climate change being imagined as “the most dire scenario”.

Later, other indirect manifestations trigger and nourish Oswego’s imagination, such as books, news articles, and YouTube videos. For example, reading about the term “Gaia” led Oswego to an “epiphany”:

2006-01-17: Seeing the world in terms of our own limited and self-inscribed boundaries, a rather narrow view of the totality of the life of our planet, we don’t see the larger picture, the way in which all life is interrelated, the way the oceans, land masses, and the atmosphere regulate the temperature of the planet and support life as we know it. […] Global warming is real, and we have pushed the envelope and made it happen, all within a few generations. Staggering when you think about it.

Oswego’s views on anthropogenic climate change underwent a fundamental shift, particularly in terms of the ingrained human exceptionalism that has defined Western-centric relations to nature (Plumwood, 1993). Reading about “Gaia” made Oswego’s view on the relation between humans and nature irrevocably intertwined. This shift in perspective echoes what Stenner calls “second-order sensemaking”; a reflexive stance on the framework that supports his sensemaking and interpretation. Oswego bemoans humans’ responsibility for altering and “push[ing] the envelope” on our ecosystem. Echoing Lovelock, Oswego writes that the “current impending crisis” gained momentum within just a few generations, emphasising the speed and scope of the climate crisis are “staggering” to think about. These indirect symbolic resources transform how Oswego views humans’ responsibility in “arrogantly attempting to conquer to earth” based on a “narrow view of the totality of life of our planet”. While these experiences do not necessarily bring the collapse temporally closer, Oswego seems to nuance his imagination of what is causing the collapse.

About six months later, the climate crisis manifests directly when a heatwave hits the city where Oswego lives, triggering apocalyptic imaginations:

2006-08-05: HEAT. But as the day dragged on in a hazy trance, the heat made even the seconds pass by in a sluggish torpor: it was a morning and night of a million little miseries, with just as many ways to get through them… Michelle O’Donnell, writing in the New York Times this week about the heat wave that has afflicted the eastern half of the country. […] Now in this fierce August heat, I am thinking there is something going on with the weather. I don’t recall it ever feeling this hot. There is something primeval, dangerous about it. I even envisioned briefly a not-so-distant planet Earth, overcome by man-made global warming, where there were wars over water and food supplies, crops regularly burned up in the fields, glaciers were almost non-existent, and people were fighting for scarce resources. I didn’t even care carry this line of thought to it’s logical conclusion. Frightening. […] I think they are harbingers of the future, and we are all going to be caught unprepared.

Directly feeling “this fierce August heat” is reinforced by an indirect symbolic resource – reading about “a hazy trance” in the New York Times. Combined, these trigger an imagination in which Oswego reflects on extreme weather and reminisces about the swelling heat in New Orleans when growing up, although he does not recall ever feeling so hot. He feels the heat is “primeval” and attributes it to the climate crisis. They also nourish a dystopian imagination of a “not-so-distant planet Earth” left ravaged – a future Oswego finds “frightening” and consciously refrains from imagining further. He describes the climate crisis as a collection of interconnected crises bundled together, hence using it as a label to coalesce various possible futures, such as food and water insecurity. The heat – directly felt and indirectly read about – triggers and nourishes a vivid imagination drawing on common apocalyptic tropes. Oswego concludes his entry by calling extreme weather events, like the heatwave, “harbingers of the future”, indicating that similar events will define the future. This move resonates with how historical analogies are used (Wagoner, this issue).

These examples relating to extreme weather events illustrate how direct and indirect manifestations are intertwined and reinforce each other. For instance, when an emotional attachment to a place intensifies the valence of a distant event, or when an article about heat and experiencing a heat wave triggers an apocalyptic imagination. While Oswego’s imagination in the early 2000 s became more concrete and apocalyptic, the collapse remains temporally distant – as something that will probably not happen in his lifetime.

### Civilizations Rise and Fall. We are Very Likely Next

A few years passed without any mention of the climate crisis, until a subtle yet direct manifestation emerged for Oswego. In the second period, we propose that his apocalyptic imagination consolidates, as the header implies, and its temporal horizon shifts much closer to the present. Oswego’s writing about the climate crisis seems to increase in frequency and the imagination becomes more differentiated. Since Oswego frequently visits parks and has a long-standing interest in taking photos of nature, it is not too surprising that unusual or abnormal seasonal changes become a manifestation of the climate crisis. He already attributed tropical rain and “summer-looking clouds” in October to global warming in 2005. When he noticed maples leafing and magnolias blooming earlier than expected, he felt both joy and despair. Oswego describes the paradox of enjoying something while remaining conscious of the looming disaster beautifully disguised in magnolias:

2013-02-06: I love seeing all this, but it is rather discomfiting also to realize that these are the effects of human-altered climate change and global warming. 2012 was the warmest year on record in the U.S. So, while I exult in the arrival of Spring in early February instead of later in the month, I also am aware of how much warmer our planet is and that the consequences of this up the road will be dire. I think from what I have read that we have passed the tipping point. It’s too late to go back and stabilize CO2 levels in the atmosphere. I know I should not dwell on these thoughts, but I have always been so sensitized to changes in the environment, especially seasonal ones, and I miss the days when we had more normal seasons and weather. I guess I am really thinking that would be back in my childhood and growing up years in the 50 s and 60s. I fear for what we have done to our earth in the name of exploiting resources, overpopulation, pollution of our air and water, and indifference to the type of world future generations will inhabit.

Others might not have thought much about blooming magnolias in February, demonstrating how the climate crisis manifests differently for each person. This direct and subtle manifestation is supported by an indirect one – the information that 2012 was the hottest year on record in the United States. These serve as symbolic resources that nourish his imagination: “the consequences of this up the road will be dire”. Oswego adds that humanity might have surpassed a tipping point, implying that a more dystopian future might now be unavoidable and, perhaps, more unpredictable – a clear shift from the evitability described in the previous period. Humans perceived indifference to the natural world and heedless consumption cement this imagination and Oswego fears for what “type of world future generations will inhabit”, still suggesting the worst fallout will happen after his lifetime.

Years down the line, Oswego describes an enchanting yet very uncommon snowfall in 2018, leaving him marvelling at the white veil and eerie silence while stressing that the snowfall represents the “unsettling fact” that it is “another byproduct of this scary thing called global warming and climate change, at least the severity of it”. Such faint breaches in temporal expectation bring the climate crisis to the fore and show how non-violent, even partly enjoyable manifestations can equally trigger imaginations. This example corresponds to the elaboration stage in Zittoun and Gillespie’s (this issue) ARETA model, in which a disruption in expectation forces people to make sense.

Oswego worked as a reporter and editor for years and has always been an avid consumer of news, which constitutes an endless stream of indirect manifestations and information about what is happening worldwide. It occasionally fosters a strong emotional response when “[he] finds himself succumbing to the awful and strange headlines”. He lists several natural and geopolitical disasters, from wildfires to wars and Trump’s first presidency, which he describes as “crazy and depressing” and reflects that “[he] reads too much”. Most concerning is the “human-caused possible end of our civilisation” – the climate crisis. News stories represent an indirect manifestation that simultaneously brings attention to the climate crisis and can have significant imaginative effects, like a CNN headline he called “the scariest headline I’ve ever read”:

2018-11-05: It’s been a little over three weeks but the shock has not worn off. It’s as if there was a time before the headline and then there is now, after the news, when the future seems much less certain and life as we know it, even just a couple of decades ahead, is emphatically in peril. Yes, we’ve been reading about it for years, and for most of us it was not something that would happen in our lifetime, or so we confidently thought. But it is happening now, each summer with heat waves and wildfires, and hurricane season and once in 500-year flooding every other year or so […] The headline is this one, and it just happened to appear in CNN online but it was all over the place: “Planet has only until 2030 to stem catastrophic climate change, experts warn” Just ponder those words for a moment. This is from a major UN report summarizing the work of thousands of scientists and many thousands of published scientific studies. […] Civilizations rise and fall. We are very likely next. […] Then just two days ago, I read that scientists had discovered that the oceans are absorbing much more heat trapped in our atmosphere by 200 years of carbon emissions than was previously thought. The oceans were helping us stave off warming disasters. Not anymore. The oceans are rapidly heating up. That means global warming will accelerate even faster. The implications of all this are beyond mind-boggling. What on earth kind of world are today’s children and young people going to inhabit? I see young couples with babies in strollers and wonder, “Do they have any idea?” It’s all rather surreal. People act like this is all just make-believe or exaggeration, or political fear mongering and “fake"science. It’s not. It’s real.

Oswego describes a “before and after” reading the headline, suggesting that he might experience a rupture. The shock persists three weeks later and marks a temporal realignment. Before reading the article, Oswego imagined the consequences of human-caused climate change to be further into the future, but now the timeline has shifted, forcing an apocalyptic climate future much closer to the present. As discussed, Oswego has been aware of the climate crisis for decades, although his climate anxiety remained subdued due to a perceived temporal distance and belief that the crisis was evitable. The information shortened the temporal horizon and strengthened the realisation that “it is happening now”. His sense of uncertainty increased and the future was now “in peril”. “[T]housands of scientists” give this reimagining legitimacy. These indirect manifestations trigger imaginations and become symbolic resources nourishing a climate future in which human civilisation might be headed for an inevitable fallout. He described the whole situation as “mind-boggling” and “surreal”, with a heightened sense of alarm and despair emerging. It is not just his own future that has been drastically reimagined but has also extended to all human civilization. The question of politics starts to feature more prominently in Oswego’s writing during this period, particularly the role of denialism. Growing political polarisation around the subject of the climate crisis also seems to impede his ability to imagine viable solutions, and Oswego fears for the next generations, who are imagined inheriting an increasingly uncertain world.

Adopting a longitudinal lens, like other papers in this special issue (Muller Mirza; Zittoun & Gillespie), illuminates the many manifestations of the climate crisis, some direct and others indirect. These vary in intensity – from subtle to violent – and in emotional tone, from enjoyable to anxious. Marvelling at blooming magnolias, for instance, contrasts with the anxiety provoked by credible reports that the crisis is advancing faster than anticipated. Together, these symbolic resources form a repertoire of experiences and information that shape Oswego’s imagination of the future.

### We have Rammed through the Tipping Point

In the last period, Oswego seems progressively convinced that humanity has passed a point of no return and uses “apocalypse” to describe the future as closer and more inevitable than ever. This usage of theological language likely reflects the vast scale of the climate crisis – something affecting everyone. Living on the eastern seaboard, Oswego has lived through plenty of hurricane seasons, and his anxiety level appears to escalate over the years. The first hurricane of 2019 is described as “the most powerful Atlantic storm in recorded history”, which Oswego views as a testimony to how humanity has “screwed up the weather and climate”. At the time, Oswego lived with and took care of his 95-year-old mother and was grateful for not receiving another evacuation note. He feared his mother would not have survived an upheaval, making the prospect even more “dreadful”. Luckily, the storm strayed from the town but triggered imaginations. The climate crisis manifests more directly for him in violent hurricane seasons, with his safety depending on the wind’s direction and therefore provoked questions of “what if it hadn’t [missed]?”. The cumulative effect (Pedersen et al., submitted) of four consecutive years of “near-misses” has heightened his anxiety and the attention he devotes to the forecast. He imagines the possibility of being “wiped off the map”, reflecting on what happened to people in the Bahamas during the hurricane that just missed his town. Oswego stresses how “planning for anything seem like an act of faith in a future more uncertain than ever”.

Following his mother’s passing in early-2020, Oswego does not have to worry about her health during a possible evacuation; however, the repeated evacuation orders and anxieties connected to the hurricanes make the question of moving away resurface:

2020-02-16: Also, after three years in a row of mandatory hurricane evacuation orders, I really don’t want to go through that anymore. Imagine if you will every August and September worrying about whether everything you own and the place you love will be literally blown off the map by a Category 5 hurricane such as Dorian, which devastated part of the Bahamas last year before heading north and veering away from us at the last minute, as predicted by the forecasts. Thank God! I had nowhere to take Mom during that evacuation, so we stayed here and prayed. That is no way to live. Granted, in the future I’ll easily be able to evacuate myself, but that’s little consolation considering what I might have to return to. As much as I’ve come to dearly love the parks and gardens here that I frequently visit, and which were saving sanctuaries for me during the time I was caring for Mom, I feel the need to begin the rest of my life someplace else.

Oswego invites the reader to take his perspective and imagine living with the recurrent prospect of destruction, and being confronted with the fragility of existence, for a few months every year. The possibility of evacuating is less frightening when not caregiving, a tension between his attachment to the place and fear of loss remains. During the time Oswego took care of his mother, most of his attention was focused on her needs and well-being. Then, when she passed, Oswego suddenly had more time to ponder the state of the world “that seems to be falling apart”, referring to the political upheaval in the United States and the climate crisis that puts “civilization at risk”. Oswego feels “greatly anguished” and recognises that his anger and dread concerning the state of the world have been boxed up whilst caregiving, whereas now “there’s no escaping it”.

Later the same year, Biden and Harris’ victory in the 2020 election renewed Oswego’s hope in the future:

2020-12-22: It’s been a tragically awful year with the pandemic, economic catastrophe, and criminals in the White House. Who would ever imagine our “democracy” would take this kind of deep dive toward oblivion. Oh, and lest we forget, amidst all the mayhem unleashed in the past four years, there’s the very real prospect of complete civilizational collapse in a matter of decades from the wholly human-caused global warming that will only increase exponentially if our leaders continue to dither with piecemeal solutions. But instead of despair, I’m going to have some hope that we can at least begin to turn the corner with a fresh start in Washington come January 20. God has shown mercy on us poor wretches who seem to be stricken with evolutionary and enlightenment dysfunction and malaise. Think about what kind of a world the youngest among us will inherit. What kind of agony will be inflicted on future generations by our stupidity, selfishness and greed? Will we wake up in time, and will the younger generations rise to the challenge of saving us from our collective folly?

Oswego imagines both apocalyptic and hopeful futures, oscillating between despair (“prospect of complete civilization collapse”) and the possibility of change (“to turn the corner with a fresh start”). The oscillation partly depends on politics since the imagined “civilisational collapse” is intrinsically linked to political (in)action and current “piecemeal solutions”. Oswego deliberately decides to maintain hope due to Biden’s promised transformation. The election acts as a symbolic resource that amplifies hope in the future. Connecting climate hope to political change highlights on one hand, the importance of government (in)decisions in how the climate crisis is being imagined and, on the other hand, how climate emotions are embedded in a politics of emotion (Ahmed, 2014). Inaction also leads Oswego to speculate what kind of world is left for “the youngest among us” and blame this future-violence on “selfishness” and capitalism, indicating that his imagination contains a moral obligation to future generations (Hourdequin, 2025). This echoes Nixon’s (2011) work on slow violence, which describe a temporal and spatial dislocation, making it difficult to hold the main culprits of climate catastrophe accountable.

When the excruciating heat returned, Oswego’s imagination further deepens in immediacy and disillusionment:

2021-07-01: Apocalypse sooner than we thought? - It’s freakishly hot in the Northwest. All-time high temperature records are being annihilated. 116 degrees in Portland. 121 in central British Columbia. Reservoirs behind dams in the West are drying up. Wildfires could be worse than last year’s. Power generation is threatened. Agriculture in California and other Western states could be decimated from lack of water. 40% of the nation’s fruits and vegetables come from that state. Our deeply flawed way of life is being exposed as never before. Greed, waste, materialism. It seems like global warming and climate change are starting to finally smack us out of our passivity as every day now we see dire forewarnings of what’s to come. It all makes me yearn to escape. But to where?

Record temperatures in the Northwest bring the climate crisis into purview in a more than indirect manner, as Oswego once lived there. Heat again triggers his imagination, steering toward an apocalyptic script using adjectives like “annihilated”, hence consolidating his sensemaking. The “all-time high temperature” resonates with other indirect manifestations, such as drying reservoirs and food shortages. For Oswego, these events expose humanity’s “deeply flawed way of life”. Yet, he also suggests that such events might break humanity’s wilful ignorance and provoke action as violent weather events increase in frequency. This is exemplified by his opening question, “[a]pocalypse sooner than we thought”, highlighting the temporal realignment that started a few years prior, shifting the imagined future from being distant and abstract to being temporally close and manifesting in very concrete ways. Arguably, Oswego is no longer shocked by what is happening, and the events act as confirmation. While the heatwave initially brought the climate crisis to the forefront, Oswego writes about a YouTube video a few days later, mentioning that if the people who are not ready for “dire truth telling” do not “wake up”, addressing the challenges will be further postponed. This illustrates how indirect manifestations become symbolic resources supplementing and differentiating his imagination. Oswego “yearns to escape” but a safe haven is nowhere in sight, suggesting that he now imagines no “outside” of the climate crisis, emphasising that neither spatial nor psychological refuges are available. Despite the temporal realignment that is consolidating, Oswego’s imagination oscillates between choosing hope (following the election) and desires of escape when realising the increased presence of heat and its potential consequences. Thus, neither hope nor anxiety are set in stone but rather shifts along his changing imagination. Hence, in 2022, Oswego’s imagination turns hopeful again after reading an essay by Rebecca Solnit published in the Guardian^1^, entitled “Ten ways to confront the climate crisis without losing hope”. Solnit proposes some avenues to deal with the climate crisis, for example, staying grounded in facts, deindividualizing the issue, remembering that the future is not immutable, and not normalising the chaos. Oswego finds solace in this essay and his imagined climate future gains a slightly more hopeful outlook, for example through techno-optimistic sentiments.

This trend of reflecting and imagining the climate crisis through the works of scientists and activists continued in 2022, when Oswego referred to “Hothouse Earth: An Inhabitant’s Guide” written by Bill McGuire.

2022-07-30: Inevitable cataclysm or not? The very possible world we face on a much more rapidly heating planet than was predicted - I think it’s the moral equivalent of a war crime… The consequences of what they’ve done are just almost unimaginable Al Gore UK climate scientist Bill McGuire finally tells the full truth that his colleagues fear to say in public […] And while all the Earth’s plant and animal species face cataclysm in decades now instead of by the end of the century, McGuire says we still have a little time left to stop the worst effects, but not much, especially since all this change is happening much more quickly than earlier computer models and estimates have predicted. […] After this summer, it’s evident we have rammed through the tipping point with our human-caused climate change, and now must act much more quickly, despite the denialists. […] The weather and climate news of the past two weeks, along with books like “Hothouse Earth” should convince even hard-core climate change deniers that they can’t keep burying their heads in the sand and driving their huge trucks, pickups, and SUVs as if they were totally free to do so, and a liveable planet be dammed.

Oswego’s imagination remains uncertain, still oscillating between apocalypse being inevitable and evitable, yet the temporal realignment remains fairly stable. While the book offers a similar message to Solnit’s essay in that there is still time to stave off the worst outcome, the tone is not as hopeful as before. The climate crisis is here and imbues everything with ethical questions. He returns to human exceptionalism and how that has justified “despoiling” the planet and believes that this should be obvious to everyone. His imagination also contains a moral component, as he writes “it’s the moral equivalent of a war crime”, referencing political (in)action and negligence. Oswego then combines these symbolic resources with more direct and palpable manifestations, such as the summer that is slowly coming to an end, acting as a signal that humanity has rammed through tipping points. Once again, the issue of denialism arises, and it appears to be part of the reason the imagined future is still bordering on the apocalypse.

In 2024, after Hurricane Helene devastated vast areas in the Appalachian Mountains, and with the storm Milton looming, Oswego wrote about the loss of lives and how his friends there tried to cope. Having watched numerous YouTube videos, he described the scene as “post-apocalyptic”. Once imagined as a retirement haven, where Oswego could sit on the porch with the Appalachian Mountains as the backdrop, it now has become a violent frontier of the climate crisis. Oswego ponders whether this will finally get the climate change deniers to take it “seriously”:

2024-10-09: They [YouTube videos] are nightmarish, surreal, and predictive of the environmental apocalypse that maybe coming sooner than any of the climate scientists expected. […] And then fatigue replaces the initial shock and disbelief. Rain events, flooding, hurricanes etc., are crazy now that human-caused global warming has truly kicked in. The scientists predicted this, but the avarice and greed of corporations associated with the fossil fuel industries led to the dangers of global warming being ignored and covered up. But we all have a portion of the blame, burning those fuels and collectively polluting and warming the planet. Did we think we could go on doing this forever? It’s such a tragedy I feel for the human race and the future when I see and hear about the destruction from Helene. It’s really depressing. No one is immune now anywhere on earth.

The videos consolidated his imagination of what the apocalyptic future might look like. In tandem with the apocalypse increasing in certainty, his emotional response also shifted from fear towards grief. He rhetorically asked, “did we think we could go on doing this forever?”, pinpointing a naïveté that had led us to where we are.

This section explored how Oswego’s temporal realignment persisted, while his imagination of a possible apocalypse grew more certain and relied on a mix of direct and indirect manifestations acting both as triggers and symbolic resources. The point of no return seemed to have been crossed. Inspired by the writings of activists and scientists, he sought to maintain hope in certain periods. However, violent weather events, an overload of dystopian predictions, and the continued greed and denial left his optimism for the future dwindling. He viewed the climate crisis as omnipresent and emphasised that “[n]o one is immune now anywhere on earth”. Oswego increasingly places responsibility on those in power, and witnessing inaction and denialism amplifies dystopian imaginations and their emotional toll.

## Conclusions

We have followed Oswego’s writing to explore where the climate crisis manifested and to trace how his imagination developed across two decades. The longitudinal and sociocultural psychological perspective applied allowed us to capture mundane and spectacular, direct and indirect, manifestations of the climate crisis, and to highlight the crisis as a continuous and contextually embedded experience. Echoing others, we assumed that the nature of the crisis might pose some imaginative challenges and across his journal, we observed a progressive scaffolding of his imagined climate future. We divided the analysis into three distinct periods, each with imaginative shifts. First, Oswego’s imagined climate future remained rather distant and abstract, and he espoused some level of eco-anxiety, yet the threat was not yet fully imagined nor imminent, instead hope seemed to coincide with evitability. He became increasingly aware of the consequences down the line. Second, Oswego began to differentiate his imagined climate future, most noticeably in its temporal realignment and growing apocalyptic undertones. He imagined civilisational collapse as increasingly plausible and as a potentiality in his lifetime, even evoking theological end-day language to describe the scale of the crisis. His eco-anxiety seemed to increase, although still fluctuating with hope. Third, Oswego’s temporal realignment persisted – if not further shifted – and humanity was imagined on an irreversible course towards an apocalypse with major tipping points passed. His imagination differentiates, for example by explicit blaming capitalism, political inaction, and denialism, and there is also a shift in eco-emotions somewhat mirroring the transformation of his imagination, moving from eco-anxiety towards eco-grief. His imagination also gained a moral dimension, discussing how humanity is failing future generations, shifting from a witnessing state to being a moral participant.

The analysis adds a more dynamic dimension to existing theories and approaches. Methodologically, we argue that the longitudinal and experiential emphasis provides unique insights into how crises are experienced and imagined over time (see also Muller Mirza, this issue). One of the major advantages is the ability to capture mundane twists and turns that are part of people’s crisis experiences and therefore to enhance sensitivity to interactions between individuals and specific characteristics of crisis. This approach is particularly important for endemic and impalpable crises because it is sensitive to manifestations in unlikely moments, events, and places over time. It redirects attention to the individual and challenges the epistemological framing of crisis as a time-bound event (Fassin, 2022; Vigh, 2008). Traditional definitions of crises tend to emphasise a moment of decision or turning point as discussed; however, these might be more suitable for crises that are sudden and spectacular but might fall short when studying more endemic and slow-building crises. In analysing Oswego’s diary, we observe that he seems to approach a more traditional understanding of crisis as time passes as he is increasingly able to identify who holds responsibility and what kinds of political action are needed.

Theoretically, we detailed how imaginations transform over two decades and propose that studying eco-emotions solely through time-bound snapshots have certain limitations. As mentioned, existing studies provide a momentary view of people’s eco-emotions toward unspecified futures, in terms of content or temporality (Pedersen, in prep.). We suggest that longitudinal designs can capture the non-linearity and complexity of emotional responses to the often impalpable climate crisis, thus providing a dynamic and contextually grounded account. For example, such studies could examine when and in what situations people experience eco-anxiety, and which form the future takes. Through this analysis, we observed that subtle changes (e.g. early spring, snowfall) provoked quieter and more reflective emotions, whereas shocks (hurricane Katrina, a CNN headline) triggered intense emotions. Over two decades, we observed how Oswego’s climate future consolidated as apocalyptic, current, and inevitable. His emotions followed this shift, oscillating between fragile hope and despair, abstract worry and embodied anxiety, ultimately transitioning from eco-anxiety towards eco-grief. The shift also echoed toward more politically mediated and morally charged emotions, for instance about intergenerational responsibility. This illustrates how eco-emotions evolve alongside imaginative shifts, becoming more concrete as the crisis becomes more immediate.

We also attempted to explore the types of manifestations to better understand the process of imagination, showing how the often-presupposed dichotomy between direct and indirect manifestations is not as straightforward as sometimes implied. In fact, establishing sharp boundaries between direct and indirect manifestations might risk oversimplifying how they are multifaceted and often entangled in everyday life. For example, the climate crisis is not just violent weather events but can manifest in subtle and non-violent weather events (e.g. early blooming magnolias) which can then be supported by scientific reports and news articles. Research could further explore the scaffolding that occurs and expand the scope beyond dramatic events to include subtle manifestations, which still impinge on imaginative processes.

While we focused on imagined futures, we also observed a reimagining of the past, tied to the realisation that humanity has crossed critical tipping points. Oswego recalls a carefree 1980 s road trip when he “wasn’t concerned about encountering unprecedented 115-degree F heat”, underscoring four decades of environmental change. His river walks, once a source of pleasure, are remembered against today’s reality: “[rivers are] drying up from prolonged record-setting heat and drought”. Such reflections reveal how the climate crisis casts past experiences in a darker light, saturating not only imagination of the future but also reinterpretation of the past.

Hoping to transcend static inquiries, we have attempted to offer a longitudinal and sociocultural argument for nuancing understandings of how people imagine and relate to climate crisis, capturing a wide range of manifestations. If imagined climate futures shift fundamentally through decades – such as from distant concern to apocalyptic urgency, from anxiety to grief, from witness to moral participant – we need more research that captures the dynamism and content of these futures. Furthermore, we chose the climate crisis as an example but also argue that this approach can be beneficial for studying crises more generally, because it allows researchers to identify which crises people experience, their characteristics and manifestations over time. Such an approach is pertinent for unearthing slower or invisible crises, which are otherwise overlooked. We hope this analysis has provided an example of how a longitudinal approach can capture one diarist’s experiences of the climate crisis and illuminate shifting levels of uncertainty in an age of ecological collapse.

## Data Availability

The data used in this study is publicly available.

## Footnotes

1
https://www.theguardian.com/environment/2021/nov/18/ten-ways-confront-climate-crisis-without-losing-hope-rebecca-solnit-reconstruction-after-covid?CMP=Share_iOSApp_Other.
